# The Use of Colors as an Alternative to Size in *Fusarium graminearum* Growth Studies

**DOI:** 10.3390/foods7070100

**Published:** 2018-06-27

**Authors:** Edgar Cambaza, Shigenobu Koseki, Shuso Kawamura

**Affiliations:** 1 Laboratory of Food Process Engineering, Graduate School of Agriculture, Hokkaido University, Sapporo 060-0808, Hokkaido, Japan; koseki@bpe.agr.hokudai.ac.jp (S.K.); shuso@bpe.agr.hokudai.ac.jp (S.K.); 2 Department of Biological Sciences, Faculty of Sciences, Eduardo Mondlane University, Av. Julius Nyerere, nr. 3453 Maputo, Moçambique

**Keywords:** *Fusarium graminearum*, mycelial growth, RGB, gray scale

## Abstract

Size-based fungal growth studies have limitations. For example, the growth in size stops in closed systems once it reaches the borders and poorly describes metabolic status, especially in the stationary phase. This might lead mycotoxin studies to unrealistic results. Color change could be a viable alternative, as pigments result from a mold’s metabolic activity. This study aimed to verify the possibility of using gray values and the RGB system to analyze the growth of *Fusarium graminearum*. It consisted of color and area measurements using ImageJ software for specimens grown in yeast extract agar (YEA). The results suggest the utility of color and gray values as reliable tools to analyze the growth of *F. graminearum*.

## 1. Introduction

Mycelial size is widely regarded as the “golden standard” for mold growth studies [1], regardless of if they are based on radius, diameter, perimeter, or area. However, this approach has some drawbacks: (1) molds do not stop growing if conditions allow; (2) the size does not tell much about the metabolism, especially in the field; (3) in closed systems, the growth in size is limited by the container and the mold takes its shape; (4) the growth in size is vulnerable to biotic or abiotic factors without explaining much about the metabolic variations; (5) it is not adequate to describe noncircular or irregular shapes; and (6) it usually overlooks the volume.

A constraint occurs when mold is grown in Petri dishes. Radius and diameter are very effective growth predictors during the lag and exponential phases. This explains how Marin et al. [2] and many other authors have been have been able to use size in their studies for many years. However, as the fungus reaches the plate’s border, it slows down the expansion and increases its thickness. It does not necessarily stop growing, probably misleading some interpretations. Indeed, Deacon [3] says that most of the secondary metabolism occurs during this period. Thus, there is a need for viable alternatives.

Color could be a good predictor of both physical and chemical changes for any organism. Little is known about this matter, but color change could be an asset to *F. graminearum* growth studies because it is a response to metabolism and maturation processes [4,5], it reflects the state of the fungus in both closed systems and field, under antagonism or any other condition [6], and, nowadays, it can be measured using accessible electronic tools.

If the colors are validated as tools to measure mold growth, they might enhance the overall quality of research on mycotoxins and other metabolites, such as antibiotics or enzymes. In the current context, if the color change is effectively demonstrated as being related to growth, it can open the possibility for researchers or farmers to know if *F. graminearum* is producing deoxynivalenol (DON) and zearalenone (ZEA) just by spotting its color.

## 2. Materials and Methods

### 2.1. Mold Isolate

This study used an *F. graminearum* isolate from the Catalogue of the Japan Collection of Microorganisms (JCM). It is registered as the teleomorph *Giberella zeae* (Schwabe) Petch, isolated by Sugiura [7] from rice stubble in Hirosaki, Aomori Prefecture, Japan. It is a known producer of deoxynivalenol, 15-acetyldeoxinivalenol and zearalenone [8].

### 2.2. Experimental Procedure

Three replicates were grown inside a chamber at room temperature on yeast extract agar (YEA) in Petri dishes. The plates were inside a black box to minimize light interference and maximize the contrast between the objects and the background. The only light was the light-emitting diode (LED) lamp from the chamber.

Daily photos from an upper view, from approximately 25 cm away, were taken using a professional Nikon D3200 camera. The photos were taken for 20 days and were used for color determination using ImageJ software (FIJI edition), developed by the National Institutes of Health [9]. The images of fungi were separated from the background using the color threshold. There were three basic measurement processes: Gray quantification, RGB (red, green and blue) analysis, and area determination. The gray scale consists of pixels representing simply the monochromatic intensity of light in the image, shown as shades of gray. ImageJ FIJI carries a native plugin to analyze the gray scale. As a predictor, it would be better than RGB because it ignores the variations in hue and would make this method suitable to analyze different mold species, as they frequently differ in color. ImageJ has a plugin called Color Histogram, available on the software’s website [9], designed to measure the RGB from pixels of a photo.

**Figure 1:**
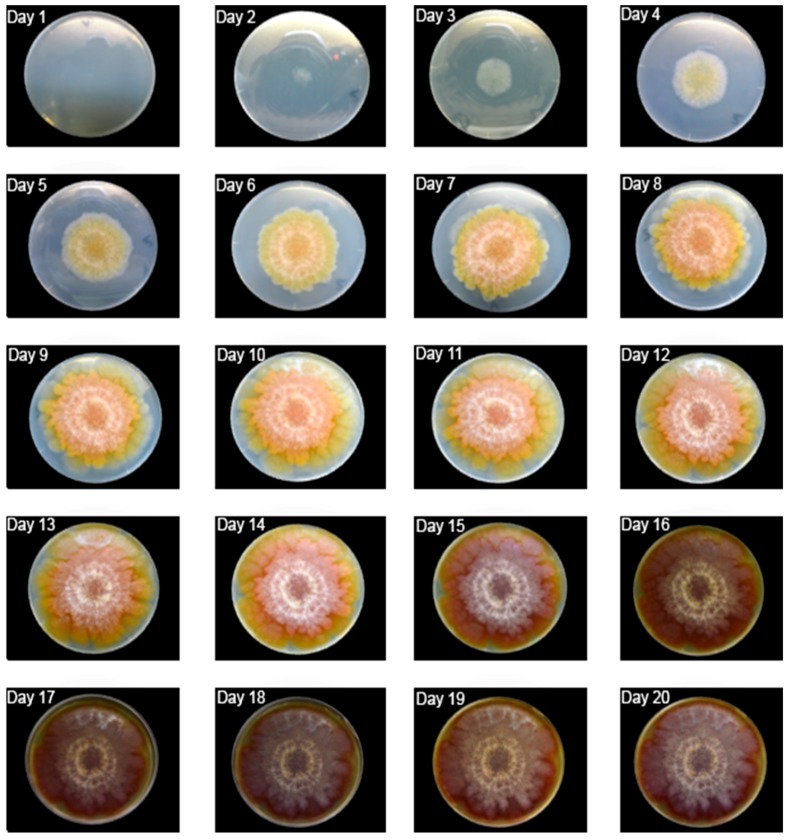
Daily growth of *F. graminearum.*

The gray scale measurements were mean, mode, minimum, maximum, skewness and kurtosis. The RGB components were analyzed through mean and mode for each color. The mycelial area was measured to validate the mean and modal gray values because it is a better-known growth variable for fungi. Mode and mean were the chosen parameters to represent the color change, as they show central tendency and are probably the most-simplified description of the phenomenon. The mean is good because it results from the input of all values, while mode is focused on the most abundant value. For the case of *F. graminearum*, it is important to pay attention to the mean because the mold’s surface has a heterogeneous color distribution (Figure 1).

Furthermore, mean might not be the best approach to analyze secondary metabolites such as mycotoxins because it includes areas with different colors, possibly different in metabolic contents and activity. Thus, mode can be a good alternative as it captures essential information and it will be particularly important if the most pigmented areas are also the ones producing more toxins.

Nevertheless, the most important are the consistency of the parameter and its ease of use in mathematical and statistical processing. For this reason, levels of dispersion of gray scale mean and modal values were compared. Additionally, the raw version of the 6-day photo was given to a panel of 21 university students with basic instructions about how to isolate the image from the background and determine the mean and mode. Several people repeated this procedure because the isolation of the photo from the background requires some personal judgment based on visual impression. The most consistent parameter was supposed to show a higher proximity between the central tendency measurements and less dispersion.

### 2.3. Statistical Analysis

The data were analyzed in StataMP, IBM SPSS Statistics 20 and Microsoft Excel. All hypotheses were tested at α = 0.05. For the gray value, mean and mode were compared using the Wilcoxon signed rank test to see if they presented significant differences. The test was performed to select the best measure of central tendency for the study. After analyzing the variations in the gray value, its skewness and kurtosis were also plotted to better analyze its distribution. Then, the gray value was correlated with the mycelium’s area.

The RGB components (mean values) were plotted and compared to trend lines through coefficients of determination (R^2^). The correlations between the different colors were also determined to see if they could be interchangeable in *F. graminearum* growth studies. The RGB channels were correlated to gray scale for the same reason.

## 3. Results and Discussion

### 3.1. Qualitative Description

At the peak of the maximum growth rate, *F. graminearum* formed a yellowish mycelium forming a gradient densely pigmented at its center. The lag phase took two days, followed by a nine-day exponential growth. It finally covered the entire surface of the Petri dish on the 11th day. From this moment, it became compact and a central reddish color expanded towards the borders.

From day 15, there was a reduction in brightness, perhaps reflecting a low metabolic rate. A white layer of mycelium increased as most of the surface changed color, becoming slightly brown.

### 3.2. Mean Versus Mode

**Figure 2:**
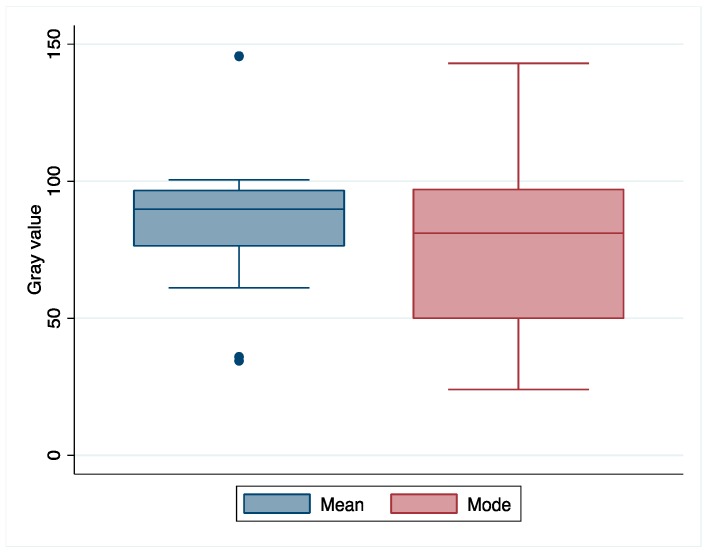
Comparison between the mean and modal gray values from the experimental data.

Figure 2 illustrates the differences between the mean and modal gray values, after the measurements based on the photo from the 6th day. The mean gray value shows a narrower range of values within the confidence interval. Indeed, the mode showed higher standard error (7.29 against 5.56) and other measures of dispersion, such as standard deviation, variance, range, and interquartile intervals. The distance between the average values and medians was 5 units for both, but the mean had higher kurtosis (2.36 against −0.019) and a narrower interquartile range (20 units) if compared to the mode (47 units). These observations suggest mean gray value as the best parameter to analyze *F. graminearum*’s brightness. As *p* = 0.014 (Wilcoxon signed rank test), it would be unwise to use both mean and mode for analysis of growth.

Similar results were observed with RGB mean and modal values from the panel of 21 students (Table 1), although it might be acceptable to interchange both parameters, considering a Wilcoxon signed rank test (*p* = 0.333).

Mode still showed a higher standard error and dispersion in general, although the kurtosis was similar for both. The distance between the central tendency measures was smaller for mean and it even had an equal median and mode (157 units).

The observations above suggest that mean is a more consistent central tendency measure than mode, and is thus a more reliable variable to analyze the growth *F. graminearum* based on colors when grown in yeast extract agar.

### 3.3. Mycelial Area and Color Measurement

**Figure 3:**
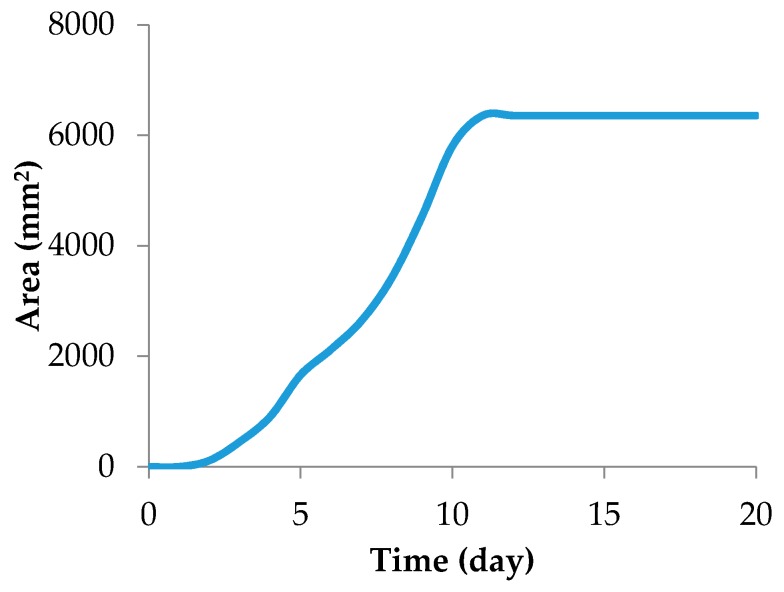
Mycelial area of *F. graminearum* for 20 days.

The mycelial area increase will be briefly described (Figure 3), as it is important to validate the color parameters as growth descriptors. The fungus grew according to the typical “s” pattern. The median lag phase lasted two days. Before that, the specimens were not observable without the aid of a microscope. Thus, major measurements started on the second day and went up to the 20th day. The exponential growth went up to the 10th day and it was possible to observe a considerable change, to a darker tone, in the overall color.

### 3.4. Gray Value Analysis

**Figure 4:**
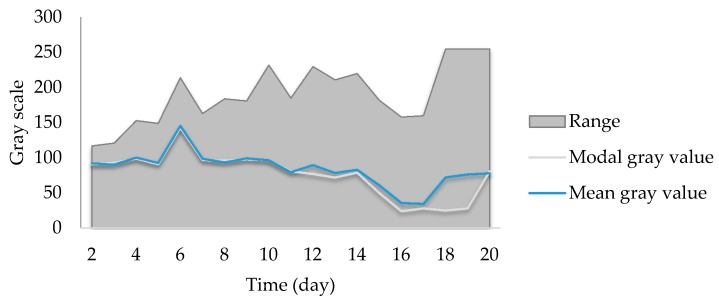
Gray value variation in the samples. The equation represents the trend line for the mean gray value.

The colors observed covered the entire range of gray scale (0 to 255) (Figure 4). The darkest tone was consistently black (0) but the maximum gray value in general increased from 117 to 255 and remained there from the 18th day. Yet, there were notable variations, such as the peaks in days 6, 10 and others, or the valley from the 16th to 17th day.

A peculiar phenomenon to consider is the daily change in slope. The brightness increased and then decreased, with only two exceptions in the final days. Unlike the maximum gray value, the central tendency measures tended to decrease globally, though the mean and mode were, as shown before, significantly different. Based on shape, they had similar trends until the 11th day (beginning of the stationary phase), when the darker tones increased in dominance. The mean was considerably consistent with a cubic function (R^2^ = 0.65); thus, it could be used to analyze the growth *F. graminearum* and perhaps mycotoxin production, especially with some increases in the degree of the polynomial.

**Figure 5:**
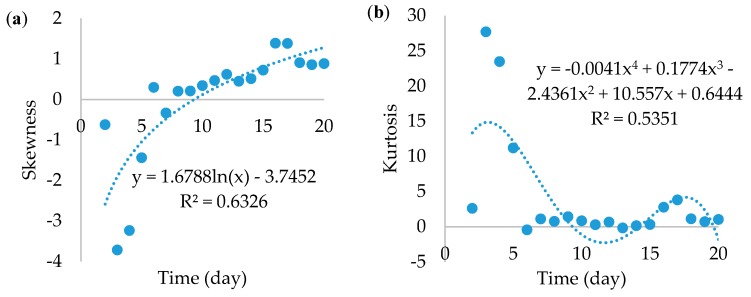
Skewness (**a**) and kurtosis (**b**) of *F. graminearum*’s gray scale.

The skewness and kurtosis of the gray scale were also analyzed (Figure 5). The skewness increased logarithmically from negative values and became positive after the 9th day. This means that, up to that day, there was a dominance of lighter colors and then there was a shift to darker ones. Such a shift might be a good milestone of the actual metabolic start of the stationary phase.

Kurtosis is a good measurement of how narrow the distribution of tones is. Its simplest acceptable fit was a 4th-degree equation. As the Figure 5b shows, there was an abundance of relatively few colors during the first days because the kurtosis was much higher than 3 (reference normal distribution), certainly up to the 6th day where there was a peak in gray values. Then, the number of colors increased drastically and remained as such until the end.

Together, skewness and kurtosis suggested a variation from few bright colors to a wide-range of darker colors. It is equally important to know the extent of gray value usability if there is a need to discuss results from previous research, considering size as the major growth property. One shall assume area as much more realistic than the radius and diameter, because it carries bi-dimensional information, especially if the mycelium is not regularly rounded.

Table 2 correlates the mean and modal gray values with the area. As shown, both variables have a significant correlation with the size at 0.01, especially the mode. This suggests the mode as the best parameter to relate with studies based on fungal area, even though mean is more appropriate to initiate and develop studies based on colors.

### 3.5. RGB Analysis

**Figure 6:**
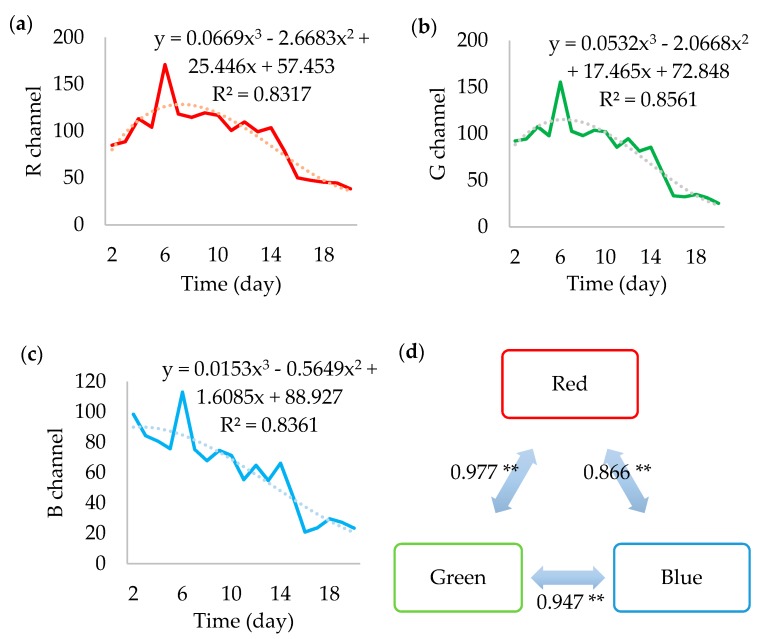
The mean values of the red (**a**), green (**b**), blue (**c**) channels, and the correlations between their means (**d**). ** Correlation is significant at the 0.01 level (2-tailed).

All RGB channels (Figure 6) showed trends similar to the gray value. Likewise, they presented the peak on the 6th day and acceptably fit to cubic functions, with an overall decline in value.

Similarly, they presented a peak on the 6th day and acceptably fit to cubic functions, with an overall decline in value. The cubic regressions resulted in R^2^ above 0.8 and it indicates the possibility of combining them in a single equation with approximately 59.5% of probability of effectively representing the simultaneous behavior of the RGB channels. Indeed, the colors seem highly correlated, meaning that some factor is causing them to change in the same fashion.

As it seems, each can be individually used to analyze the growth of *F. graminearum*, but the green component showed the best performance. However, they cannot replace each other, as Friedman’s two-way analysis showed (*p* < 0.001). In more advanced analyses, each color might be more appropriated to study a particular chemical or metabolic phenomenon. Until then, it is preferable to include all and separately.

### 3.6. How Related Are the Gray and RGB Measurements?

As the Table 3 shows, all colors are correlated to gray parameters and once again green shows stronger relationship. The choice of gray scale to analyze color is convenient as it only consists of one parameter. However, it neglects the hue and saturation, certainly related to some phenomena contributing to pigmentation. Furthermore, gray scale might be convenient to easily extrapolate observations between species, as even fungi from the same genera might substantially differ in terms of color, especially hue. On the other hand, the colors, especially green, seem more consistent.

Unlike most of the results shown above (except for the correlation with area), the modal gray value shows more affinity with the mean RGB values. This observation might require more attention in the future, as some property of the colors was somehow better evidenced through abundance, even in the absence of hue and saturation. If the modal gray value showed a higher correlation with mean RGB values and area, it was probably more accurate than mean, though less precise.

## 4. Conclusions

It is possible to use color to analyze and predict the growth of *F. graminearum* during the first 20 days in yeast extract agar. The color parameters showed algebraically predictable behaviors, either when observed throughout *F. graminearum*’s growth or if related to radius, a well-known growth variable. Even a simple observation shows a gradual change, which is easy to correlate to several biological phenomena. This approach can, in the future, be extended to other species or groups of molds, considering their own color variations. It would be useful to develop a detailed catalog of color variations of different fungi containing their mathematical descriptions through their lifecycles.

However, colors do not necessarily behave like size, although it is possible to correlate the color change and the fungal area. Thus, it is necessary to develop new models rather than retrieving the already-reasonably-designed method for radius or diameter. In the future, it will also be important to standardize the method to ensure its reproducibility. For instance, it is important to harmonize the type of camera or lens used to photograph the mold, the growth medium, register the exact settings for light, temperature, angle and height at which the photos are taken, among other factors potentially affecting the results.

Color analysis is very promising, as it keeps changing even in the stationary phase. Indeed, the fungus increases in the number of colors at this stage, though it also becomes very dark. This possibility to verify color change will certainly provide a better understanding of fungal metabolism than simple size measurements.

## Figures and Tables

**Table 1:** Comparison between the mean and modal colors (RGB) from a 6-day photo of *F. graminearum* measured by 21 different people.

Statistics	Mean	Mode
Average	147.89	146.71
Standard error	3.10	3.62
Median	157	160
Mode	157	173
Standard-deviation	24.61	28.74
Variance	605.77	825.98
Kurtosis	−1.52	−1.52
Skewness	−0.53	−0.61
Range	63	66
Minimum	111	107
Maximum	174	173
Sum	9317	9243
Count	63	63

**Table 2:** Correlation between the gray central tendency measures and mycelial area of *F. graminearum*.

	Parameter	Area (mm^2^)
Mean gray value	Pearson Correlation	−0.533 *
	Sig. (2-tailed)	0.019
	N	19
Modal gray value	Pearson Correlation	−0.577 **
	Sig. (2-tailed)	0.010
	N	19

* Correlation is significant at the 0.05 level (2-tailed). ** Correlation is significant at the 0.01 level (2-tailed).

**Table 3:** Correlations between RGB colors and the central tendency gray measures.

Correlations		Mean Gray Value	Modal Gray Value
Red	Pearson Correlation	0.825 **	0.865 **
	Sig. (2-tailed)	<0.001	<0.001
	N	19	19
Green	Pearson Correlation	0.858 **	0.902 **
	Sig. (2-tailed)	<0.001	<0.001
	N	19	19
Blue	Pearson Correlation	0.846 **	0.881 **
	Sig. (2-tailed)	<0.001	<0.001
	N	19	19

** Correlation is significant at the 0.01 level (2-tailed).

## References

[1] Garcia D., Ramos A.J., Sanchis V., Marin S. (2009). Predicting mycotoxins in foods: A review. Food Microbiol..

[2] Marin S., Cuevas D., Ramos A.J., Sanchis V. (2008). Fitting of colony diameter and ergosterol as indicators of food borne mould growth to known growth models in solid medium. Int. J. Food Microbiol..

[3] Deacon J.W. (2006). Fungal Biology.

[4] Suhr K.I., Haasum I., Steenstrup L.D., Larsen T.O. (2002). Factors affecting growth and pigmentation of penicillium caseifulvum. J. Dairy Sci..

[5] Wong H.C., Koehler P.E. (1981). Production and isolation of an antibiotic from monascus purpureus and its relationship to pigment production. J. Food Sci..

[6] Nasuno S., Asai T. (1961). Red pigments formation by interaction of molds. J. Gen. Appl. Microbiol..

[7] Sugiura Y., Microorganisms J.C.O. (1996). Gibberella zeae (schwabe) petch. JCM Catalogue.

[8] Sugiura Y., Watanabe Y., Tanaka T., Yamamoto S., Ueno Y. (1990). Occurrence of gibberella zeae strains that produce both nivalenol and deoxynivalenol. Appl. Environ. Microbiol..

[9] Schneider C.A., Rasband W.S., Eliceiri K.W. (2012). Nih image to imagej: 25 years of image analysis. Nat. Methods.

